# Cocaine Self-administration and Extinction Inversely Alter Neuron to Glia Exosomal Dynamics in the Nucleus Accumbens

**DOI:** 10.3389/fncel.2019.00581

**Published:** 2020-01-10

**Authors:** Rachel Jarvis, Alessandra Tamashiro-Orrego, Vanessa Promes, Leona Tu, Jinyuan Shi, Yongjie Yang

**Affiliations:** ^1^Department of Neuroscience, Tufts University School of Medicine, Boston, MA, United States; ^2^Graduate School of Biomedical Sciences, Tufts University, Boston, MA, United States

**Keywords:** cocaine, exosome, astroglia, GLT1, microglia

## Abstract

Alteration of glutamatergic synaptic plasticity in the Nucleus Accumbens (NAc) has been implicated in cocaine-seeking behaviors. Astroglial mechanisms for maintaining extracellular glutamate homeostasis through cysteine/glutamate exchanger (xCT) and glutamate transporter GLT1 are dysregulated following cocaine exposure and contribute to altered glutamatergic synaptic plasticity. However, how these astroglial proteins become dysregulated in cocaine addiction remains unknown. We recently showed that neuron to astroglial exosome signaling is essential to maintain GLT1 protein expression by transferring neuronal miR-124-3p into astrocytes to suppress GLT1-inhibiting microRNAs (miRs) in astrocytes. In the current study, by selectively labeling neuronal exosomes using CD63-GFP^f/+^ exosome reporter mice, we examined how the self-administration and extinction stages of the mouse cocaine self-administration model alter neuronal exosome signaling to astrocytes and microglia in the NAc. We found that cocaine (but not food) self-administration strongly reduces the internalization of neuronal exosomes, particularly in astrocytes in the NAc (but not in motor cortex), which can be effectively reversed by extinction training. In parallel, cocaine self-administration alone specifically and differentially affects activation of glial cells by decreasing GFAP expression in astrocytes but increasing Iba1 expression in microglia. However, extinction training fully reverses the increased Iba1 expression in microglia but only partially reverses the reduction of GFAP in astrocytes. Taken together, our study reveals altered *in vivo* dynamics of NAc neuronal exosomes in the cocaine addiction model, providing new insights about how altered neuron to glial exosome signaling may contribute to astroglial dysfunction in cocaine addiction.

## Introduction

Cocaine addiction is a chronic neurological disorder primarily manifested with compulsive drug-seeking behaviors, which are coordinately influenced by multiple neural circuitries in different brain regions, including at least prefrontal cortex, amygdala, nucleus accumbens (NAc), and ventral tegmental area (VTA; Kelley, 2004; Kalivas, 2009). In particular, NAc receives and integrates inputs from both cortical and limbic regions and is considered a central brain structure in developing reward-driven (including drug-seeking) behaviors (Scofield et al., 2016a). Early observations have shown altered dopaminergic neurotransmission as an underlying mechanism for drug-seeking behavior (Berridge and Robinson, 1998). Cocaine-mediated inhibition of dopamine transporter (DAT) leads to elevation of extracellular dopamine levels that enhance dopaminergic synaptic transmission in NAc, resulting in a strengthened rewarding effect of drug-seeking behavior (Nestler, 2005).

Recently, altered glutamate homeostasis and glutamatergic synaptic plasticity in NAc has also been implicated in cocaine addiction behavior (Kalivas, 2009). Cocaine addiction affects both neuronal and astroglial mechanisms for maintaining proper glutamate homeostasis. Cocaine decreases activity and protein expression of the cysteine/glutamate exchanger xCT that is enriched in NAc astrocytes for extracellular transport of glutamate (Baker et al., 2002; Madayag et al., 2007; Knackstedt et al., 2010), leading to reduced activation of pre-synaptic mGluR2/3 receptors and enhanced presynaptic glutamate release (Moussawi and Kalivas, 2010). Cocaine also decreases functional uptake of extracellular glutamate-mediated by the predominant glutamate transporter GLT1 (Knackstedt et al., 2010; Trantham-Davidson et al., 2012), further contributing to increased extracellular glutamate levels. Restoration of xCT or GLT1 expression by N-acetylcysteine (NAC) or the β-lactam antibiotic ceftriaxone, respectively, is sufficient to inhibit cocaine- and cue-induced reinstatement of drug-seeking behavior (Baker et al., 2003; Knackstedt et al., 2010; Reichel et al., 2011). However, how xCT and GLT1 become dysregulated in cocaine addiction remains unknown.

Although GLT1 (human analog EAAT2) is predominantly and abundantly expressed in adult astrocytes across the CNS, the physiological induction of GLT1 in astrocytes is strongly dependent upon neuronal signals (Yang et al., 2009). By employing a VGluT1 KO and generating mGluR5 conditional knock-out mice, we previously showed that glutamatergic neuronal signaling, presumably glutamate itself, plays a role in the developmental induction of GLT1 (Morel et al., 2014). Recently, we also showed that cultured neurons secrete exosomes, a major class of extracellular vesicles (EVs) with a size range of 40–100 nm that originate from endosomes, that contain abundant miR-124 that can be internalized into astrocytes to increase miR-124 levels in astrocytes (Men et al., 2019). MicroRNAs (miRs) are a class of non-coding RNAs with a length of 20–15 nucleotides that actively and significantly modulate development and disease progress in many tissues including the CNS. MiR-124 is one of the most abundant neuronal miRs in the CNS, which is essential for neuronal differentiation during early embryogenesis by antagonizing the anti-neural REST/SCP1 pathway (Conaco et al., 2006). MiR-124 is also an active modulator of synaptic connectivity and plasticity (McNeill and Van Vactor, 2012). Neuronal exosomes and miR-124 are also able to significantly up-regulate GLT1 protein levels by suppressing endogenous GLT1-inhibiting miRs in astrocytes (Men et al., 2019). EVs and exosomes secreted from various CNS cell types have emerged as a novel and important intercellular communication pathway in the CNS that has also been implicated in pathological conditions of the CNS, including neurological injury (Xiong et al., 2017), neurodegenerative diseases (Quek and Hill, 2017), and glioma (Gourlay et al., 2017). Whether exosome signaling between neurons and glia is altered in cocaine addiction has not been explored. In the current study, we examined the *in vivo* dynamics of neuronal exosomes using our recently developed exosome reporter (CD63-GFP^f/f^) mice in the cocaine self-administration model.

## Materials and Methods

### Animals

The CD63-GFP^f/f^ mice have been previously published (Men et al., 2019), and were generated in house with help by Biocytogen (Worcester, MA, USA). CamKIIα-CreERT transgenic mice (strain #012362) were obtained from the Jackson Laboratory. CD63-GFP^f/f^ and CaMKIIα-CreERT mice were bred to generate the experimental CamKIIα-CreERT^+^CD63-GFP^f/+^ mice. Bac *aldh1l1*-eGFP mice were obtained from the GENSAT project through the Jackson Laboratory. Both male and female mice were used for all experiments. All mice were housed on a 12 h light/dark cycle (lights on at 07:00) with food and water provided *ad libitum*. Care and treatment of the mice during all procedures strictly adhered to the NIH Guide for the Care and Use of Laboratory Animals, the Guidelines for the Use of Animals in Neuroscience Research, and the Tufts University IACUC policies.

### Drugs and Tamoxifen Injection

Cocaine HCl (20 mg/ml) was generously provided by the NIDA Drug Supply Program and diluted to working concentration in sterile saline. Tamoxifen (4-OHT, Sigma) was resuspended in 100% ethanol at 20 mg/ml, and then diluted to 2 mg/ml in sunflower oil for injection. CamKIIα-CreERT^+^CD63-GFP^f/+^ mice were given a single 10 mg/kg dose of tamoxifen by intraperitoneal (i.p.) injection at postnatal day 42 (P42).

### Catheter Implantation Surgery

Mice were anesthetized with isoflurane (induction 3–5%, maintenance 1–3%) for surgical implantation of an indwelling jugular vein catheter connected to a head-mounted entry port, as previously described (Griffin et al., 2007). Briefly, the right jugular vein was exposed, and the catheter tubing was inserted and secured to the vein. The catheter was passed under the skin to an incision on the head, and the port was secured to the skull with dental cement anchored by screws. Mice were given buprenorphine (0.1 mg/kg) for analgesia and allowed to recover for 3–7 days before beginning self-administration. Catheter patency was maintained by daily infusion of 100 μl heparinized saline (100 U/μl) through the catheter. Catheter patency was confirmed by infusion of 20–30 μl Brevital (methohexital, 10 mg/ml) through the catheter the day before self-administration training began, after the last self-administration session, and as needed during self-administration training if mice did not acquire self-administration behavior or if their behavior changed unexpectedly. Mice that did not show signs of sedation within 2–3 s of Brevital infusion were excluded from the study as having non-patent catheters.

### Behavior Training

All behavior testing was done in extra wide mouse operant chambers with a modified top (Med Associates, VT, USA). The chambers were fitted with a touchscreen on one side, which was covered with an aperture plate to frame the two presented images. The opposite side of the chamber contained a food pellet receptacle connected to a pellet dispenser, with a stimulus light above the pellet receptacle. The chambers also contained a house light and a tone generator. Cocaine was delivered by a syringe pump located outside the chamber, and the drug delivery tubing was connected to the mice through a swivel and swivel arm (Instech, Plymouth Meeting, PA, USA) mounted on top of the chamber to allow free movement of the animals during self-administration. All aspects of the sessions were controlled and recorded by K-Limbic software (Conclusive Marketing, Limited, UK).

### Cocaine Self-administration

Mice were not pre-trained with food to press the touchscreen and were not food-restricted during the study. Self-administration sessions took place once daily during the light cycle for 12 consecutive days. Two images, a circle and an X were presented on the touchscreen (white images on black background, 2 cm × 2 cm). The X was considered the active image and the circle of the inactive image for all mice. The side the active image was presented on was counterbalanced within groups. The availability of cocaine reward was indicated by illumination of the house light and displaying the images on the touchscreen. Touching the active image resulted in a 2 s infusion of cocaine (1 mg/kg in a volume of 12–30 μl, based on weight). Each infusion was paired with a cue (1 s tone and illumination of the stimulus light) followed by a 30 s time-out signaled by turning off the house light and removing the images from the touchscreen. Pressing the inactive image was recorded but had no consequences. Sessions lasted 2 h, or until mice received 30 infusions (30 mg/kg) of cocaine (to prevent overdose). For the first 3 days of self-administration training, a non-contingent dose of cocaine was given through the jugular catheter using the software controls at the start of the session, as well as at intervals throughout the session, to facilitate acquisition of self-administration behavior (no more than eight non-contingent infusions per session, <4 non-contingent infusions/session on average). Sham mice were implanted with a catheter and placed in the operant chambers with identical images and cues, but were not connected to the syringe pump and received no reward for pressing the active image. Sham mice experienced 2–4 non-contingent activations of the paired light and sound cues (as if the active image had been pressed) using the software controls, for the first 3 days of self-administration training. Mice met criteria for acquisition of self-administration when they: (1) self-administered at least 20 infusions in a session; (2) >70% of responses were on the active image; and (3) had no more than 20% variation in number of infusions between sessions.

### Cocaine Extinction

Two days following the last self-administration session, extinction training began. Extinction sessions took place 6 days per week. The same active and inactive images (on the same side as during self-administration) were presented on the touchscreen, and the house light was kept turned off. Pressing the touchscreen on either the active or inactive image had no consequences, but all touches were recorded. Mice met criteria for extinction when they: (1) made fewer than eight responses on the active image; and (2) did not distinguish between the active and inactive image (<70% of responses on active image). All mice met extinction criteria within the 18–20 total days of extinction training.

### Food Self-administration

Mice in the food self-administration group were not implanted with a jugular catheter and were not food-restricted prior to or during behavior training. Food self-administration took place 5 days per week and was carried out as close to cocaine self-administration procedures as possible. The same active and inactive images were used, but pressing the active image resulted in the delivery of a food pellet (14 mg dustless precision pellets, Bio-Serv) along with the paired light and sound cues. The time-out period was a minimum of 30 s (as in cocaine self-administration), but mice additionally had to retrieve the food pellet (detected by a head entry sensor in the pellet receptacle) before the next trial could start. To facilitate acquisition of food self-administration during the first 1–4 sessions, non-contingent food reward was delivered (once at the start of the session and at intervals during the session, no more than eight non-contingent rewards/session) and trials were manually advanced if the mouse did not retrieve pellet within ~10 min. Food self-administration sessions ended after 2 h, or when mice received 50 food pellets. Mice in the sham group were placed in the operant chambers under the same conditions, except that no food reward was delivered for pressing the active image. Mice met food self-administration criteria when they: (1) self-administered at least 30 food pellets in a session; (2) >70% of responses were on the active image; and (3) had no more than 20% variation in number of infusions between sessions.

### Tissue Processing, Immunohistochemistry, and Imaging

Two hours after the last behavior session, animals were deeply anesthetized with ketamine (100 mg/kg) + xylazine (10 mg/kg) in saline by intraperitoneal (i.p.) injection. Mice were perfused intracardially with ice-cold 4% paraformaldehyde (PFA) in PBS. Brains were dissected out and post-fixed overnight in 4% PFA at 4°C, then cryoprotected in 30% sucrose for 48 h at 4°C. The brains were embedded and frozen in OCT Compound Tissue-Tek^®^ (Sakura, Tokyo Japan). Twenty micrometer coronal sections were prepared using a Leica HM525 cryostat and mounted on glass SuperFrost+ slides (Thermo Fisher Scientific). For immunohistochemistry, slides were rinsed in PBS then treated with blocking buffer (1% BSA, 5% goat serum, 0.2% Triton in 1× PBS) for 1 h at room temperature. Primary antibodies against GFAP (1:1,000, Dako) and Iba1 (1:500, Wako) were incubated overnight at 4°C in blocking buffer. After washing slides three times in PBS, the secondary antibody (1:2,000, goat anti-rabbit AlexaFluor 633) was added for 2 h at room temperature. The slides were rinsed three times in PBS before mounting with ProLong Gold Antifade Mountant with DAPI (Thermo Fisher Scientific). Representative whole-brain image was taken at 10× magnification using the Keyence BZ-X710 Slide Scanner. Confocal images were taken using the Nikon A1R confocal laser scanning microscope. Representative high magnification images were taken with the 40× (numerical aperture 1.3) objective (15–20 μm *z*-stack with 0.5 μm step). Images for quantification were taken with a 20× (numerical aperture 0.75) objective (13–20 μm *z*-stack with 1.5 μm step). Confocal imaging settings and thresholding parameters were kept consistent within each behavioral group (self-administration, self-administration followed by extinction, and food self-administration).

To quantify the number of CD63-GFP puncta overlapped with astrocytes or microglia, a maximum intensity projection (MIP) image of the GFAP or Iba1 image was first generated. The GFAP or Iba1 labeled glia were then outlined using the freehand tool in ImageJ to create a region of interest (ROI). Next, a MIP image of the CD63-GFP puncta (green channel) was generated, and the image was converted to 8-bit grayscale and then thresholded to remove the background. The glia ROI was then overlaid on the thresholded CD63-GFP image, and the particle analysis function in ImageJ was used to analyze the number of CD63-GFP puncta inside the glia ROI. The area of the ROI was measured, and the number of CD63-GFP puncta was divided by the ROI area to find the number of puncta per μm^2^ of glia ROI. GFAP and Iba1 intensity were measured by first generating a MIP image, then converting the image to 8-bit grayscale, and finally using the Measure function in ImageJ to determine the average intensity. Microglia were counted from the MIP of the Iba1 channel. Images from BAC *aldh1l1*-eGFP mice were used to count astrocytes in the NAc, based on the MIP of the GFP channel. DAPI signal was overlaid whenever counting cells to ensure that only bona fide cells were counted.

For immunohistochemistry analyses (puncta count per glia ROI, GFAP/Iba1 intensity, microglia counts), four mice per group were used for cocaine and sham self-administration and cocaine and sham extinction; for food self-administration, three mice were used, and for sham food self-administration, two mice were used. For analysis of CD63-GFP puncta inside of glia, 20 astrocytes or microglia were counted per animal. For counting the number of astrocytes in NAc after cocaine or sham self-administration, three mice per group were used.

### Experimental Design and Statistical Analysis

Sample sizes and the statistical approach used for each experiment are described in the figure legends. Previous results were used to estimate reasonable sample sizes. All analyses were performed using GraphPad Prism 7. All values were plotted as column graphs with individual values shown or as a cumulative probability curve. For graphs with error bars, error bars are presented as SEM. No custom code was used in the analysis. For two-group comparison, an unpaired two-tailed student’s *t*-test was used. For cumulative probability curves comparison, the Kolmogorov–Smirnov test was used. Behavior data were analyzed using a two-way repeated-measures ANOVA. Statistical significance was tested at a 95% (*P* < 0.05) confidence level, *P*-values are shown in each graph, and *F*-scores (F), *t*-scores (t), and degrees of freedom (*df*) are reported in the text and figure legends. Outlier analysis was done in Prism using the ROUT method (*Q* = 1%), and individual data points identified as outliers were removed.

## Results

**Figure 1:**
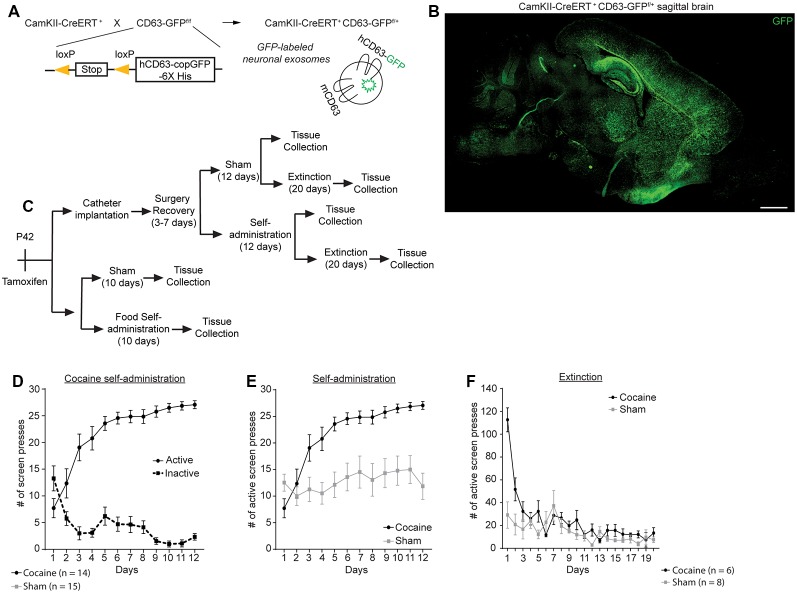
Cocaine self-administration and extinction in CamKIIα-CreER^+^ CD63-GFP^f/+^ mice. **(A)** Schematic diagram of the breeding strategy to generate neuron-specific exosome/intraluminal vesicle (ILV) reporter mice. **(B)** Representative image showing distribution of CD63-GFP puncta in the brain of CamKIIα-CreER^+^CD63-GFP^f/+^ mice. Scale bar: 2 mm. **(C)** Schematic showing the experimental strategy and behavior testing used in this study. **(D)** Active and inactive image screen presses for cocaine self-administration group; *n* = 14 mice, significant main effect of image (*F*_(1,26)_ = 94.44, *p* < 0.0001) and time (*F*_(11,286)_ = 3.064, *p* = 0.007), as well as significant interaction time × image (*F*_(11,286)_ = 14.56, *p* < 0.0001). **(E)** Cocaine and sham self-administration of mice used in this study; total number of active screen presses are shown; *n* = 14 mice (cocaine group), *n* = 16 mice (sham group), significant main effect of group (*F*_(1,27)_ = 16.62, *p* = 0.0004) and time (*F*_(11,297)_ = 12.41, *p* < 0.0001, as well as a significant interaction time × group (*F*_(11,297)_ = 6.605, *p* < 0.0001). **(F)** Extinction of cocaine and sham mice following self-administration; *n* = 6 (cocaine group), *n* = 8 sham group. Statistics calculated using two-way repeated measures ANOVA.

We recently generated exosome reporter CD63-GFP^f/f^ mice in which GFP-fused CD63 (membrane marker of exosomes) can be induced following Cre-dependent recombination (Men et al., 2019). To selectively label neuronal exosomes *in vivo*, we bred CD63-GFP^f/f^ mice with CaMKIIα-CreER^+^ mice that specifically express Cre recombinase in neurons under the CaMKIIα promoter, to generate CaMKIIα-CreER^+^CD63-GFP^f/+^ mice (Figure 1A). Following a single injection of 4-OHT (i.p., 10 mg/kg) at P42, clear and robust expression of the CD63-GFP reporter throughout the cortex, hippocampus, and striatum were observed (Figure 1B). Adult (P120–140) CaMKIIα-CreER^+^CD63-GFP^f/+^ mice were implanted with an indwelling jugular catheter, as described previously (Griffin et al., 2007) and split into different groups for the behavior training (Figure 1C). One group of mice was used for cocaine self-administration training only (“Self-administration”), while another group went through both cocaine self-administration and extinction training before tissue collection (“Extinction”). Sham mice were also implanted with a catheter and placed in the operant chambers with identical images and cues as the cocaine groups, but were not connected to the syringe pump and received no reward for pressing the active image during either the self-administration or the extinction phase. During the cocaine self-administration training, CaMKIIα-CreER^+^CD63-GFP^f/+^ mice with access to cocaine clearly favored the active image over the inactive image on the touchscreen [Figure 1D; 2-way repeated measures ANOVA showed significant main effect of image (*F*_(1,26)_ = 94.44, *p* < 0.0001) and time (*F*_(11,286)_ = 3.064, *p* = 0.007), as well as significant interaction time × image (*F*_(11,286)_ = 14.56, *p* < 0.0001)], while the sham group (no cocaine access) did not distinguish between the active and inactive images (data not shown). As a result, the CamKIIα-CreER^+^CD63-GFP^f/+^ mice that underwent cocaine self-administration pressed the active image on the touchscreen significantly more than the sham CamKIIα-CreER^+^CD63-GFP^f/+^ mice (Figure 1E; 2-way repeated measures ANOVA showed significant main effect of group (*F*_(1,27)_ = 16.62, *p* = 0.0004) and time [*F*_(11,297)_ = 12.41, *p* < 0.0001, as well as a significant interaction time × group (*F*_(11,297)_ = 6.605, *p* < 0.0001)]. The mice in the cocaine group self-administered, on average, 24–28 mg/kg of cocaine daily during days 5–12 of self-administration training. During extinction training, the mice that had undergone cocaine self-administration exhibited an initial burst of a high number of presses on the active image but quickly decreased to a low level of touches on the active image, while the sham group remained at a baseline low level of active image touches (Figure 1F). We observed that the use of a touchscreen for this operant behavior paradigm sometimes resulted in false-positive pressing of the touchscreen in the sham group (25% of mice) but not in the cocaine group, due to mice either laying down or perching next to the touchscreen with their flank or tail in contact with the screen, which can lead to unusually high numbers of screen presses (>100). To minimize the interference of these false-positive presses of the touchscreen, we performed outlier analysis (see “Materials and Methods” section for details) and removed identified outliers (Figure 1F, <1% of data points in cocaine group, 7% of data points in sham group).

**Figure 2:**
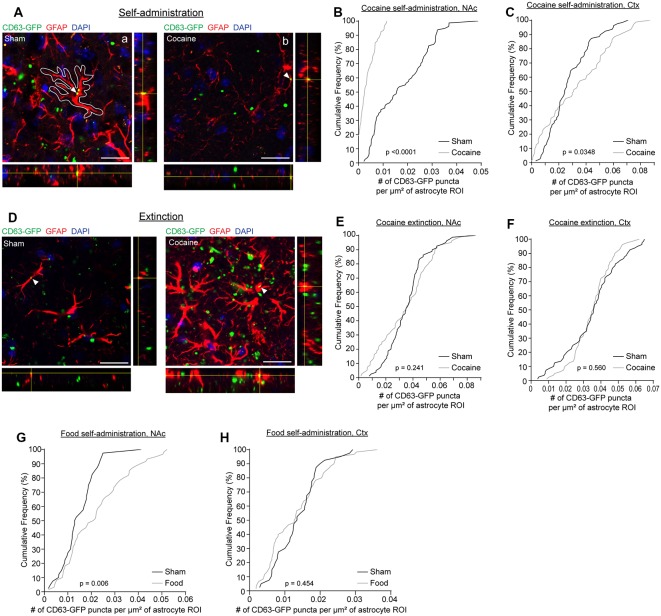
Cocaine self-administration reduces the overlap of CD63-GFP puncta with astrocytes. **(A)** Representative images showing an overlap of CD63-GFP puncta with astrocyte GFAP staining in sham **(a)** and cocaine **(b)** self-administration mice. Each image shows a single slice from a *z*-stack along with the orthogonal view. White outline in the image **(a)** is representative of the ROIs used to measure total CD63-GFP puncta within individual astrocytes. Scale bar: 20 μm. Cumulative frequency curves showing number of GFP puncta per μm^2^ of astrocyte ROI after sham or cocaine self-administration in the nucleus accumbens (NAc; **B**, *p* < 0.0001), or the cortex (**C**, *p* = 0.0348); *n* = 20 cells/mouse, four mice/group. **(D)** Representative images showing an overlap of CD63-GFP puncta with astrocyte GFAP staining in sham and cocaine extinction mice. Each image shows a single slice from a *z*-stack along with the orthogonal view. Scale bar: 20 μm. Cumulative frequency curves showing number of GFP puncta per μm^2^ of astrocyte ROI after sham or cocaine extinction in the NAc (**E**, *p* = 0.241) or the cortex (**F**, *p* = 0.560); *n* = 20 cells/mouse, four mice/group. Cumulative frequency curves showing number of GFP puncta per μm^2^ of astrocyte ROI after sham or food self-administration in the NAc (**G**, *p* = 0.006) or cortex (**H**, *p* = 0.454); *n* = 20 cells/mouse, two mice in the sham group, three mice in food self-administration group. *P*-values determined by the Kolmogorov–Smirnov test.

We have previously shown that neuron-derived CD63-GFP^+^ exosomes can be taken up by astrocytes *in vitro* and *in vivo* (Morel et al., 2013; Men et al., 2019). We have also shown that neuron-derived exosomes carry miRNA cargo that can up-regulate important astrocyte protein expression and functions, such as the glutamate transporter GLT1 and glutamate uptake function (Men et al., 2019). Cocaine self-administration and extinction is known to significantly down-regulate GLT1 expression (Knackstedt et al., 2010; Fischer et al., 2013), which contributes to dysregulation of glutamate homeostasis in the NAc and drug-seeking synaptic plasticity. To determine whether there are alterations in neuron to astrocyte exosomal signaling, we examined whether neuron-derived CD63-GFP^+^ exosomes were differentially taken up by astrocytes following cocaine self-administration training. We focused on NAc, one of the central regions involved in cocaine addiction, and motor cortex, a control region that has not been closely associated with cocaine addiction (Cooper et al., 2017). GFAP immunostaining was performed to identify individual astrocytes in cocaine and sham self-administration CaMKIIα-CreER^+^CD63-GFP^f/+^ mice. Although GFAP clearly labels individual astrocytes in the NAc and co-localization of neuronal exosomes (indicated by CD63-GFP) with GFAP immunoreactivity was observed (Figures 2A,D), as GFAP immunoreactivity only labels major branches but not fine processes of astrocyte morphology (Morel et al., 2014), we then used the GFAP immunoreactivity as a guide to determine individual astrocyte domains (as shown in the white outline in Figure 2Aa) to include the portion of astrocyte morphology that is not labeled by GFAP staining. We subsequently quantified the number of CD63-GFP puncta inside selected astrocyte domains using the ImageJ region of interest (ROI) approach and divided the number of puncta by the area of the astrocyte ROIs. After self-administration training, 90% of astrocytes in NAc of the cocaine group have 0.01 or fewer CD63-GFP puncta per μm^2^ inside GFAP-based astrocyte ROIs (a representative image in Figure 2Ab), while at least 60% of astrocytes in NAc of the sham group have more than 0.01 CD63-GFP puncta per μm^2^ inside the GFAP based ROIs and 20% have more than 0.03 CD63-GFP puncta per μm^2^ (Figure 2B). In the cortex, however, there is a slightly higher number of puncta per μm^2^ of astrocytes after cocaine self-administration compared to the sham group (Figure 2C), suggesting a NAc-specific reduction of astroglial internalization of neuronal CD63-GFP^+^ exosomes. This reduction also appears to be specifically induced by cocaine self-administration, as extinction training completely reversed the reduction of astroglial internalization of neuronal CD63-GFP^+^ exosomes in the NAc (Figure 2E). The distribution of the average number of CD63-GFP puncta per μm^2^ inside GFAP-based astrocyte ROIs is very comparable between the sham (NAc: 0.0359 ± 0.0016 puncta/μm^2^; ctx: 0.0351 ± 0.0017 puncta/μm^2^) and the cocaine extinction (NAc: 0.0347 ± 0.0021 puncta/μm^2^; ctx: 0.0348 ± 0.0012 puncta/μm^2^) groups in both NAc and cortex (Figures 2E,F).

Cocaine self-administration procedures involve both the drug cocaine itself and the learning paradigm. To determine whether the cocaine itself or the learning paradigm contributes to the altered neuronal exosome dynamics in astrocytes, we additionally quantified CD63-GFP puncta inside GFAP-based astrocyte ROIs in food self-administration or sham training groups. This also allowed us to determine whether a natural reward would have the same effect on astroglial internalization of neuronal CD63-GFP^+^ exosomes as the drug reward, cocaine. We found no difference in the number of CD63-GFP puncta per μm^2^ of astrocyte ROIs between the sham and food self-administration groups in the cortex (Figure 2H; sham: 0.0138 ± 0.0009 puncta/μm^2^; cocaine: 0.0132 ± 0.0010 puncta/μm^2^), and in fact saw a slight increase in the number of CD63-GFP puncta per μm^2^ of astrocyte ROIs in NAc after food self-administration training (Figure 2G; sham: 0.0148 ± 0.0012 puncta/μm^2^; food: 0.0221 ± 0.0017 puncta/μm^2^). This further suggests that the reduction in astroglial internalization of neuronal CD63-GFP^+^ exosomes is region (NAc but not cortex) and stimulus (cocaine but not food) specific, and thus is not likely to be due to a general effect of reward-motivated operant learning.

**Figure 3:**
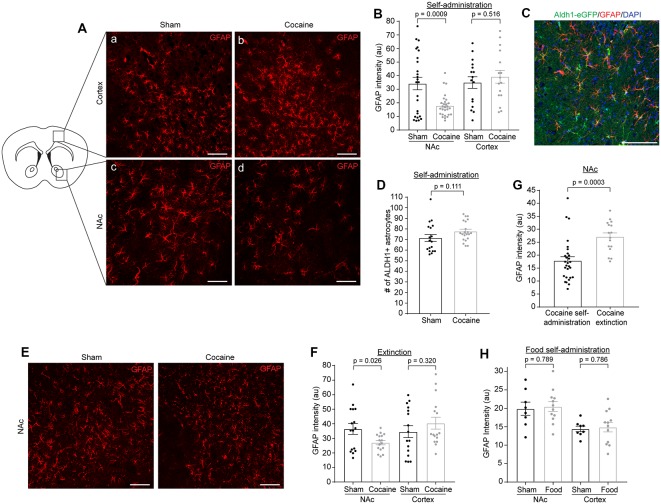
GFAP immunofluorescence is reduced in NAc after cocaine self-administration and is partially rescued after extinction training. **(A)** Representative images showing GFAP immunofluorescence in the cortex **(a,b)** and NAc **(c,d)** after sham or cocaine self-administration. The diagram shows the regions of cortex and NAc that were imaged. Scale bar: 100 μm. **(B)** Quantification of GFAP immunofluorescence intensity in NAc (*p* = 0.0009, *t* = 3.541, *df* = 51) and cortex (*p* = 0.516, *t* = 0.657, *df* = 30) after sham or cocaine self-administration; *n* = 4–12 images/mouse, four mice (total 16–28 images)/group. **(C)** Representative image showing overlap of *aldh1l1*-eGFP astrocyte reporter with GFAP staining. Scale bar: 100 μm. **(D)** Quantification of *aldh1l1*-eGFP^+^ astrocytes in NAc after sham or cocaine self-administration (*p* = 0.111, *t* = 1.631, *df* = 37); *n* = 6–7 images/mouse, three mice/group. **(E)** Representative images showing GFAP immunofluorescence in the NAc after sham or cocaine extinction. Images were taken from same region as the diagram in **(A)**. Scale bar: 100 μm. **(F)** Quantification of GFAP immunofluorescence intensity in NAc (*p* = 0.026, *t* = 2.338, *df* = 30) and cortex (*p* = 0.320, *t* = 1.01, *df* = 30) after sham or cocaine extinction; *n* = 4 images/mouse, four mice/group. **(G)** Comparison of GFAP immunofluorescence intensity in NAc after cocaine self-administration and cocaine extinction (*p* = 0.0003, *t* = 3.91, *df* = 42); *n* = 4–12 images/mouse, four mice (16–28 images)/group. **(H)** Quantification of GFAP immunofluorescence intensity in NAc (*p* = 0.789, *t* = 0.278, *df* = 18) and cortex (*p* = 0.786, *t* = 0.275, *df* = 18) after sham or food self-administration; *p* = 4 images/mouse, two mice in sham group, three mice in food self-administration group. *P*-values, *t* scores, and degrees of freedom determined by two-tailed unpaired *t*-test.

Although several addictive drugs (opioids, methamphetamine) tend to activate astrocytes in the brain (Beitner-Johnson et al., 1993; Friend and Keefe, 2013), evidenced by increased GFAP immunoreactivity, how cocaine specifically affects astrocyte reactivity has not been well documented, especially at different stages of the cocaine self-administration paradigm or in different delivery models. In fact, one study showed that GFAP expression in the NAc is reduced after cocaine self-administration and extinction (Scofield et al., 2016b), while another study, using i.p injection, found increased GFAP immunoreactivity in the dentate gyrus (Fattore et al., 2002). We performed GFAP immunostaining following cocaine self-administration and found that GFAP immunoreactivity was dramatically reduced (Figures 3Ac,d,B, *p* = 0.0009, *t* = 3.541, *df* = 51) in the NAc in cocaine self-administration mice compared to sham mice. This GFAP reduction appears to be specific to the NAc, as we found no differences in GFAP immunoreactivity in the cortex (Figures 3Aa,b,B; sham: 34.97 ± 4.29, cocaine: 39.17 ± 4.74). To determine whether this reduction in GFAP immunoreactivity represented a decrease in the number of astrocytes, we used BAC-*aldh1l1*-eGFP reporter mice, which express eGFP under the control of the *aldh1l1* promoter, to label astrocytes (Cahoy et al., 2008). We then performed GFAP immunostaining on the NAc sections and quantified eGFP^+^ astrocytes from BAC-*aldh1l1*-eGFP mice following cocaine or sham self-administration training. As shown in Figure 3C, GFAP immunoreactivity that overlapped with eGFP was clearly visualized in individual astrocytes. Despite the reduced GFAP immunoreactivity in the NAc after cocaine self-administration, we found no difference in the number of eGFP^+^ astrocytes in the NAc between sham and cocaine self-administration mice (Figure 3D, *p* = 0.111, *t* = 1.631, *df* = 37; sham: 71.50 ± 3.33, cocaine: 77.67 ± 2.03), supporting the notion that the overall number of astrocytes in the NAc is not affected, while their GFAP immunoreactivity is significantly decreased by cocaine self-administration. To determine whether the extinction process influences GFAP expression in NAc astrocytes, we further examined GFAP immunoreactivity (representative images in Figure 3E) after cocaine self-administration and extinction training. Although GFAP immunoreactivity levels in the NAc are still lower in cocaine extinction trained mice compared to sham extinction trained mice (Figure 3F, *p* = 0.026, *t* = 2.338, *df* = 30), they are significantly higher than GFAP immunoreactivity levels in mice that have only undergone cocaine self-administration training (Figure 3G, *p* = 0.0003, *t* = 3.91, *df* = 42), indicating that extinction training partially reverses the significant cocaine self-administration-induced reduction of GFAP immunoreactivity in NAc astrocytes. On the other hand, GFAP immunoreactivity in the cortex was comparable between the sham (34.56 ± 4.09) and cocaine extinction (40.40 ± 4.09) groups (Figure 3F). Interestingly, food self-administration training had no effect on GFAP intensity in either NAc or cortex (Figure 3H), suggesting that the reduction in GFAP immunoreactivity in NAc astrocytes is specifically induced by cocaine, and is not a general consequence of reward-based learning.

**Figure 4:**
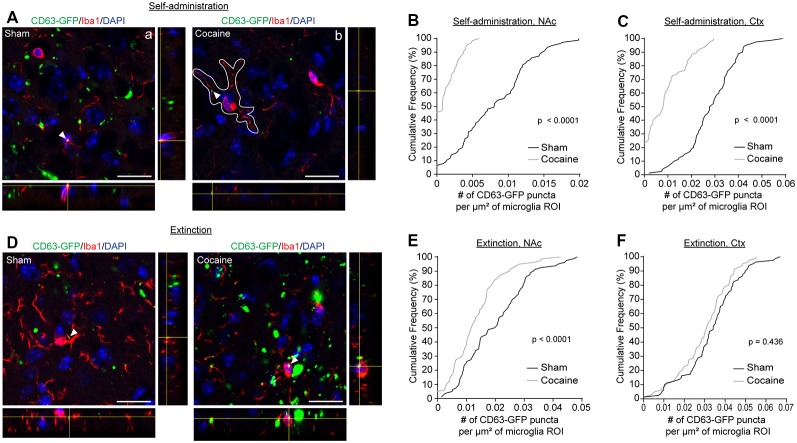
Cocaine self-administration reduces the overlap of CD63-GFP puncta with microglia. **(A)** Representative images showing an overlap of CD63-GFP puncta with microglia Iba1 staining in NAc of sham **(a)** and cocaine **(b)** self-administration mice. Each image shows a single slice from a *z*-stack along with the orthogonal view. White outline in image **(b)** is representative of the ROIs used to measure total CD63-GFP puncta within microglia. Scale bar: 20 μm. Cumulative frequency curves showing number of GFP puncta per μm^2^ of microglia ROI after sham or cocaine self-administration in NAc (**B**, *p* < 0.0001) or cortex (**C**, *p* < 0.0001); *n* = 20 cells/mouse, four mice/group. **(D)** Representative images showing an overlap of CD63-GFP puncta with microglia Iba1 staining in NAc of sham and cocaine extinction mice. Scale bar: 20 μm. Cumulative frequency curves showing number of GFP puncta per μm^2^ of microglia ROI after sham or cocaine extinction in NAc (**E**, *p* = 0.0001) or cortex (**F**, *p* = 0.436); *n* = 20 cells/mouse, four mice/group. *P*-values determined by the Kolmogorov–Smirnov test.

As the major phagocytic cell type in the CNS, microglia are known to quickly clear debris and foreign pathogens. Recent studies have shown that microglia are able to engulf secreted exosomes and facilitate the spreading of phosphorylated tau and α-synuclein aggregates (Alvarez-Erviti et al., 2011; Asai et al., 2015). To determine whether cocaine induces alterations in neuron to microglial exosome signaling, we examined microglial internalization of neuronal CD63-GFP^+^ exosomes in the NAc of CaMKIIα-CreER^+^CD63-GFP^f/+^ mice following cocaine self-administration training alone or followed by extinction training. High magnification confocal images (Figures 4A,D) showed co-localization between Iba1 immunostaining signals and CD63-GFP^+^ puncta in both sham (Figure 4Aa) and cocaine (Figure 4Ab) self-administration or extinction mice. To quantify the average number of CD63-GFP^+^ puncta taken up by microglia, we used the Iba1 immunoreactivity as a guide, in the same way as we used the GFAP immunoreactivity for the astrocytes (Figure 2Aa), to define individual microglial regions of interest (ROIs, Figure 4Ab). We then determined the number of CD63-GFP^+^ puncta per μm^2^ of the microglial ROIs. Cocaine self-administration substantially reduced the number of CD63-GFP^+^ puncta inside the Iba1-based microglia ROIs in both the NAc (Figure 4B; *p* = 0.0001) and the cortex (Figure 4C; *p* < 0.0001) when compared to sham mice. In both examined regions, it is also noted that the percentage of microglia that have no co-localized neuronal CD63-GFP^+^ exosomes increased significantly (from <10 to 45% in the NAc and from 0 to ~25% in the cortex), suggesting a completely diminished neuronal exosomal signaling in these microglia. Interestingly, extinction training differentially affects the number of CD63-GFP puncta overlapped with microglia ROIs between the sham and cocaine group in the NAc and in the cortex. While microglia still tend to have a significantly lower number of overlapped CD63-GFP puncta per μm^2^ of microglia ROI in the cocaine extinction group compared to the sham extinction group in the NAc (Figure 4E, *p* = 0.0001), there was no significant difference in the number of CD63-GFP puncta per μm^2^ of microglia ROIs overlapped with microglia ROIs between the sham (0.0335 ± 0.0015 puncta/μm^2^) and cocaine (0.0301 ± 0.0014 puncta/μm^2^) group in the cortex after extinction training (Figure 4F).

**Figure 5:**
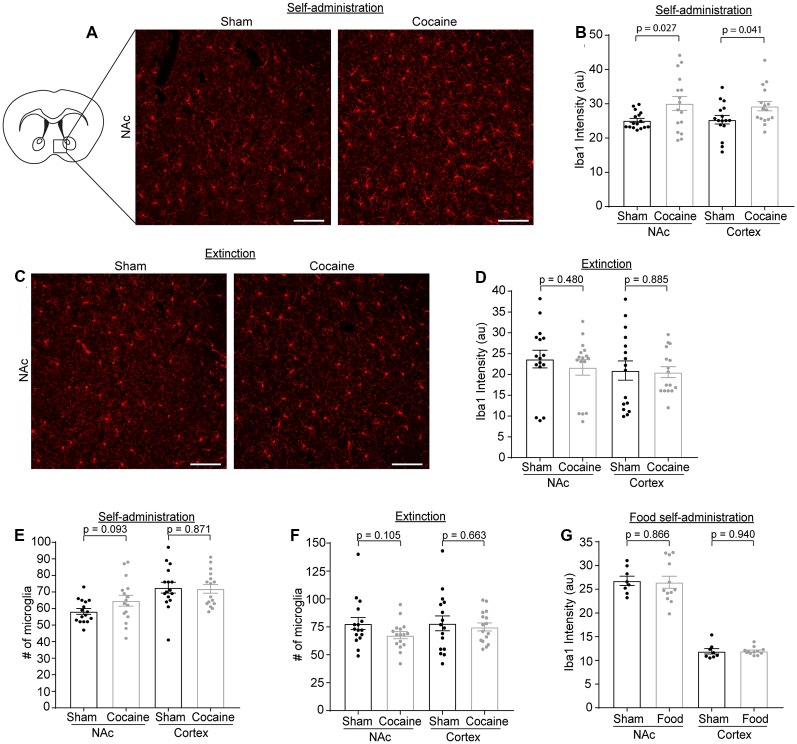
Iba1 immunoreactivity is increased after cocaine self-administration, but not after extinction training or food self-administration. **(A)** Representative images showing Iba1 immunofluorescence in the NAc after sham or cocaine self-administration. The diagram shows the region of NAc that was imaged. Scale bar: 100 μm. **(B)** Quantification of Iba1 immunofluorescence intensity in NAc (*p* = 0.027, *t* = 2.329, *df* = 30) and cortex (*p* = 0.041, *t* = 2.13, *df* = 30) after sham or cocaine self-administration; *n* = 4 images/mouse, four mice/group. **(C)** Representative images showing Iba1 immunofluorescence in the NAc after sham or cocaine extinction. Images were taken from same region as the diagram in **(A)**. Scale bar: 100 μm. **(D)** Quantification of Iba1 immunofluorescence intensity in NAc (*p* = 0.480, *t* = 0.715, *df* = 30) and cortex (*p* = 0.885, *t* = 0.145, *df* = 30) after sham or cocaine extinction; *n* = 4 images/mouse, four mice/group. **(E)** Quantification of the number of Iba1^+^ microglia in NAc (*p* = 0.093, *t* = 1.737, *df* = 30) and cortex (*p* = 0.871, *t* = 0.164, *df* = 30) after sham or cocaine self-administration; *n* = 4 images/mouse, four mice/group. **(F)** Quantification of the number of Iba1^+^ microglia in NAc (*p* = 0.105, *t* = 1.669, *df* = 30) and cortex (*p* = 0.663, *t* = 0.440, *df* = 30) after sham or cocaine extinction; *n* = 4 images/mouse, four mice/group. **(G)** Quantification of Iba1 immunofluorescence intensity in NAc (*p* = 0.866, *t* = 0.171, *df* = 18) and cortex (*p* = 0.940, *t* = 0.076, *df* = 18) after sham or food self-administration; *n* = 4 images/mouse, two mice in sham group, three mice in food self-administration group. *P*-values, *t*-scores, and degrees of freedom determined by two-tailed unpaired *t*-test.

Microglia have been shown to be activated in response to cocaine exposure (Lacagnina et al., 2017). We determined Iba1 immunoreactivity as well as the number of microglia through Iba1 immunostaining at each stage of the cocaine or food self-administration behavior paradigm. Representative Iba1 staining confocal images are shown in Figure 5A, from which we noticed an increase of Iba1 immunoreactivity after cocaine self-administration. Subsequent quantification confirmed that Iba1 immunoreactivity was increased in both NAc (Figure 5B, *p* = 0.027, *t* = 2.329, *df* = 30) and cortex (Figure 5B, *p* = 0.041, *t* = 2.13, *df* = 30) after cocaine self-administration, consistent with previous observations that microglia become activated following cocaine exposure. We also examined Iba1 immunoreactivity in the NAc and cortex after extinction training in both the sham and cocaine group (Figure 5C). The cocaine self-administration-induced increase in Iba1 immunoreactivity was completely rescued by extinction training in both the NAc and cortex (Figure 5D). Despite the increased Iba1 immunoreactivity, there was no difference in the overall number of microglia, quantified based on positive Iba1 staining, in either NAc or cortex after sham or cocaine self-administration, or extinction (Figures 5E,F). The increase of Iba1 levels but not the microglial numbers suggests that cocaine likely induces transient activation of microglia that is reversed once mice become extinguished from responding for cocaine. As a control, we also examined Iba1 immunoreactivity following food self-administration and found no differences in Iba1 immunoreactivity in either NAc (sham—26.79 ± 0.98, food—26.49 ± 1.27) or cortex (sham—11.91 ± 0.57, food—11.95 ± 0.23, Figure 5G).

## Discussion

In the current study, by selectively labeling neuronal exosomes using CD63-GFP^f/+^ exosome reporter mice, we examined how the self-administration and extinction stages of the mouse cocaine self-administration paradigm alter neuronal exosome signaling to astrocytes and microglia in the NAc. We found that cocaine (but not food) self-administration strongly reduces the internalization of neuronal exosomes in both glial types, which can be effectively reversed by extinction training. Our results also showed that cocaine self-administration itself sufficiently and substantially reduces GFAP expression in the NAc, in contrast to observations that addictive drugs (morphine, methamphetamine, and alcohol) often activate astrocytes by increasing their GFAP expression in the NAc and other brain regions.

Exosomal signaling is emerging as a new mode of intercellular communication in the CNS that has been implicated in development, metastasis of glioblastoma, stroke, and disease spreading of neurodegenerative diseases (Budnik et al., 2016). Although most of the current understanding of exosome signaling is derived from *in vitro* studies, we recently developed the cell-type-specific CD63-GFP^f/+^ exosome reporter mice for examining cell-type-specific exosome signaling changes *in vivo* (Men et al., 2019). Because exosomes are derived from endosomal intraluminal vesicles (ILVs) and released from multivesicular bodies (MVBs; Colombo et al., 2014), CD63-GFP also labels these intracellular organelles, which are typically significantly larger (>250 μm) than exosomes. In our quantification, we specifically quantified CD63-GFP signals within the GFAP or Iba1-based astrocyte or microglia ROI, as these CD63-GFP puncta are initially derived from neurons (with the use of the neuron-specific CaMKIIα-Cre) and are internalized into glial cells, thus representing secreted neuronal exosomes. It is noted that the small exosome size may not be fully resolved with confocal microscopy, which may result in multiple small puncta being quantified as a single particle by this quantification method. However, as all experimental groups were quantified under the same conditions, the results should still be insightful to show how cocaine alters neuronal exosome signaling to glial cells.

There is growing evidence to suggest the involvement of astrocytes and microglia in modulating cocaine addiction behaviors (Lacagnina et al., 2017). In particular, astrocyte-mediated regulation of extracellular glutamate levels through xCT and GLT1 is a key mechanism in modifying synaptic plasticity in the NAc that is related to cocaine-seeking (Kalivas, 2009). Although several studies have observed down-regulation of GLT1 following cocaine exposure (Knackstedt et al., 2010; Fischer-Smith et al., 2012; Fischer et al., 2013), how GLT1 becomes dysregulated remains unknown. Our current study observed a significantly reduced internalization of neuronal exosomes in NAc astrocytes. We recently showed that a subset of miRNAs are abundantly packed in neuronal exosomes and these miRNAs, especially miR-124-3p, can be transferred into astrocytes to increase protein expression of GLT1 (Men et al., 2019). In particular, *in vivo* inhibition of miR-124-3p or exosome secretion is sufficient to decrease GLT1 protein levels and glutamate uptake (Men et al., 2019). Therefore, cocaine-induced reduction of the internalization of neuronal exosomes into astrocytes could result in less transferred miR-124-3p into astrocytes and consequently decreased GLT1 expression. This process appears to be cocaine and self-administration specific, as food self-administration has no effect on the internalization of neuronal exosomes in astrocytes and cocaine extinction reversed the reduced internalization of neuronal exosomes in astrocytes. In parallel, it has been reported that several neuronal miRs, including miR-124-3p, are down-regulated by cocaine exposure (Cabana-Dominguez et al., 2018). It is thus conceivable that the reduced miR-124 signaling, including reduced transfer from neurons to astrocytes through exosomes, underlies cocaine-induced down-regulation of GLT1 expression.

Although glial cells are often activated following addictive drug exposure, our results found that GFAP expression is significantly decreased. This is an intriguing observation as reduced GLT1 is often associated with reactive astrocytes with highly increased GFAP levels in injury and degenerative conditions. However, reduced GLT1 and GFAP expression have been associated with several psychiatric disease models, including anxiety, stress, or depression (Banasr et al., 2010; Imbe et al., 2012; Gunn et al., 2013). It is likely that astrocytes undergo different molecular changes in psychiatric conditions vs. injury/degenerative conditions. GFAP alone can be inadequate and limiting to indicate the reactivity change of astrocytes in these conditions. As astrocyte-specific or single cell-based transcriptome techniques become more available (Morel et al., 2017; Zeisel et al., 2018), these approaches are likely to gain more comprehensive insights as to how astrocytes are altered in response to cocaine, which may provide new insights about their involvement in modulating cocaine addiction behaviors.

## Data Availability Statement

No large datasets were generated from this study. The data that support the findings of this study are available from the corresponding author upon reasonable request.

## Ethics Statement

The animal study was reviewed and approved by Tufts University Institutional Animal Care and Use Committee (IACUC).

## Author Contributions

RJ performed catheterization surgeries, behavioral testing, immunostaining, and confocal imaging. AT-O and VP performed behavioral testing, immunostaining, and confocal imaging. LT and JS performed immunostaining and confocal imaging. RJ, AT-O, VP, LT and JS also performed data analysis. RJ co-wrote the manuscript. YY designed the overall study, analyzed the data, and wrote the manuscript.

## Conflict of Interest

The authors declare that the research was conducted in the absence of any commercial or financial relationships that could be construed as a potential conflict of interest.

## References

[B1] Alvarez-Erviti L. Seow Y. Schapira A. H. Gardiner C. Sargent I. L. Wood M. J.. (2011). Lysosomal dysfunction increases exosome-mediated alpha-synuclein release and transmission. Neurobiol. Dis. 42, 360–367. 10.1016/j.nbd.2011.01.029 21303699 PMC3107939

[B2] Asai H. Ikezu S. Tsunoda S. Medalla M. Luebke J. Haydar T.. (2015). Depletion of microglia and inhibition of exosome synthesis halt tau propagation. Nat. Neurosci. 18, 1584–1593. 10.1038/nn.4132 26436904 PMC4694577

[B3] Baker D. A. McFarland K. Lake R. W. Shen H. Toda S. Kalivas P. W. (2003). N-acetyl cysteine-induced blockade of cocaine-induced reinstatement. Ann. N. Y. Acad. Sci. 1003, 349–351. 10.1196/annals.1300.023 14684458

[B4] Baker D. A. Xi Z. X. Shen H. Swanson C. J. Kalivas P. W. (2002). The origin and neuronal function of *in vivo* nonsynaptic glutamate. J. Neurosci. 22, 9134–9141. 10.1523/jneurosci.22-20-09134.2002 12388621 PMC6757683

[B5] Banasr M. Chowdhury G. M. Terwilliger R. Newton S. S. Duman R. S. Behar K. L.. (2010). Glial pathology in an animal model of depression: reversal of stress-induced cellular, metabolic and behavioral deficits by the glutamate-modulating drug riluzole. Mol. Psychiatry 15, 501–511. 10.1038/mp.2008.106 18825147 PMC3347761

[B6] Beitner-Johnson D. Guitart X. Nestler E. J. (1993). Glial fibrillary acidic protein and the mesolimbic dopamine system: regulation by chronic morphine and Lewis-Fischer strain differences in the rat ventral tegmental area. J. Neurochem. 61, 1766–1773. 10.1111/j.1471-4159.1993.tb09814.x 8228992

[B7] Berridge K. C. Robinson T. E. (1998). What is the role of dopamine in reward: hedonic impact, reward learning, or incentive salience? Brain Res. Brain Res. Rev. 28, 309–369. 10.1016/s0165-0173(98)00019-8 9858756

[B8] Budnik V. Ruiz-Canada C. Wendler F. (2016). Extracellular vesicles round off communication in the nervous system. Nat. Rev. Neurosci. 17, 160–172. 10.1038/nrn.2015.29 26891626 PMC4989863

[B9] Cabana-Dominguez J. Arenas C. Cormand B. Fernandez-Castillo N. (2018). MiR-9, miR-153 and miR-124 are down-regulated by acute exposure to cocaine in a dopaminergic cell model and may contribute to cocaine dependence. Transl. Psychiatry 8:173. 10.1038/s41398-018-0224-5 30166527 PMC6117282

[B10] Cahoy J. D. Emery B. Kaushal A. Foo L. C. Zamanian J. L. Christopherson K. S.. (2008). A transcriptome database for astrocytes, neurons and oligodendrocytes: a new resource for understanding brain development and function. J. Neurosci. 28, 264–278. 10.1523/JNEUROSCI.4178-07.2008 18171944 PMC6671143

[B11] Colombo M. Raposo G. Thery C. (2014). Biogenesis, secretion and intercellular interactions of exosomes and other extracellular vesicles. Annu. Rev. Cell Dev. Biol. 30, 255–289. 10.1146/annurev-cellbio-101512-122326 25288114

[B12] Conaco C. Otto S. Han J. J. Mandel G. (2006). Reciprocal actions of REST and a microRNA promote neuronal identity. Proc. Natl. Acad. Sci. U S A 103, 2422–2427. 10.1073/pnas.0511041103 16461918 PMC1413753

[B13] Cooper S. Robison A. J. Mazei-Robison M. S. (2017). Reward circuitry in addiction. Neurotherapeutics 14, 687–697. 10.1007/s13311-017-0525-z 28324454 PMC5509624

[B14] Fattore L. Puddu M. C. Picciau S. Cappai A. Fratta W. Serra G. P.. (2002). Astroglial *in vivo* response to cocaine in mouse dentate gyrus: a quantitative and qualitative analysis by confocal microscopy. Neuroscience 110, 1–6. 10.1016/s0306-4522(01)00598-x 11882367

[B15] Fischer K. D. Houston A. C. Rebec G. V. (2013). Role of the major glutamate transporter GLT1 in nucleus accumbens core versus shell in cue-induced cocaine-seeking behavior. J. Neurosci. 33, 9319–9327. 10.1523/jneurosci.3278-12.2013 23719800 PMC3694387

[B16] Fischer-Smith K. D. Houston A. C. Rebec G. V. (2012). Differential effects of cocaine access and withdrawal on glutamate type 1 transporter expression in rat nucleus accumbens core and shell. Neuroscience 210, 333–339. 10.1016/j.neuroscience.2012.02.049 22433294 PMC3358423

[B17] Friend D. M. Keefe K. A. (2013). Glial reactivity in resistance to methamphetamine-induced neurotoxicity. J. Neurochem. 125, 566–574. 10.1111/jnc.12201 23414433 PMC3637419

[B18] Gourlay J. Morokoff A. P. Luwor R. B. Zhu H. J. Kaye A. H. Stylli S. S. (2017). The emergent role of exosomes in glioma. J. Clin. Neurosci. 35, 13–23. 10.1016/j.jocn.2016.09.021 27771233

[B19] Griffin W. C. III Randall P. K. Middaugh L. D. (2007). Intravenous cocaine self-administration: individual differences in male and female C57BL/6J mice. Pharmacol. Biochem. Behav. 87, 267–279. 10.1016/j.pbb.2007.04.023 17561241 PMC2692891

[B20] Gunn B. G. Cunningham L. Cooper M. A. Corteen N. L. Seifi M. Swinny J. D.. (2013). Dysfunctional astrocytic and synaptic regulation of hypothalamic glutamatergic transmission in a mouse model of early-life adversity: relevance to neurosteroids and programming of the stress response. J. Neurosci. 33, 19534–19554. 10.1523/jneurosci.1337-13.2013 24336719 PMC3858624

[B21] Imbe H. Kimura A. Donishi T. Kaneoke Y. (2012). Chronic restraint stress decreases glial fibrillary acidic protein and glutamate transporter in the periaqueductal gray matter. Neuroscience 223, 209–218. 10.1016/j.neuroscience.2012.08.007 22890077

[B22] Kalivas P. W. (2009). The glutamate homeostasis hypothesis of addiction. Nat. Rev. Neurosci. 10, 561–572. 10.1038/nrn2515 19571793

[B23] Kelley A. E. (2004). Memory and addiction: shared neural circuitry and molecular mechanisms. Neuron 44, 161–179. 10.1016/j.neuron.2004.09.016 15450168

[B24] Knackstedt L. A. Melendez R. I. Kalivas P. W. (2010). Ceftriaxone restores glutamate homeostasis and prevents relapse to cocaine seeking. Biol. Psychiatry 67, 81–84. 10.1016/j.biopsych.2009.07.018 19717140 PMC2795043

[B25] Lacagnina M. J. Rivera P. D. Bilbo S. D. (2017). Glial and neuroimmune mechanisms as critical modulators of drug use and abuse. Neuropsychopharmacology 42, 156–177. 10.1038/npp.2016.121 27402494 PMC5143481

[B26] Madayag A. Lobner D. Kau K. S. Mantsch J. R. Abdulhameed O. Hearing M.. (2007). Repeated N-acetylcysteine administration alters plasticity-dependent effects of cocaine. J. Neurosci. 27, 13968–13976. 10.1523/jneurosci.2808-07.2007 18094234 PMC2996827

[B27] McNeill E. Van Vactor D. (2012). MicroRNAs shape the neuronal landscape. Neuron 75, 363–379. 10.1016/j.neuron.2012.07.005 22884321 PMC3441179

[B28] Men Y. Yelick J. Jin S. Tian Y. Jarvis R. Chiang R.. (2019). Exosome reporter mice reveal the involvement of exosomes in mediating neuron to astroglia communication in the CNS. Nat. Commun. 10:4136. 10.1038/s41467-019-11534-w 31515491 PMC6742670

[B29] Morel L. Higashimori H. Tolman M. Yang Y. (2014). VGluT1+ neuronal glutamatergic signaling regulates postnatal developmental maturation of cortical protoplasmic astroglia. J. Neurosci. 34, 10950–10962. 10.1523/jneurosci.1167-14.2014 25122895 PMC4131010

[B30] Morel L. Regan M. Higashimori H. Ng S. K. Esau C. Vidensky S.. (2013). Neuronal exosomal miRNA-dependent translational regulation of astroglial glutamate transporter GLT1. J. Biol. Chem. 288, 7105–7116. 10.1074/jbc.m112.410944 23364798 PMC3591620

[B31] Morel L. Chiang M. S. R. Higashimori H. Shoneye T. Iyer L. K. Yelick J.. (2017). Molecular and functional properties of regional astrocytes in the adult brain. J. Neurosci. 37, 8706–8717. 10.1523/jneurosci.3956-16.2017 28821665 PMC5588463

[B32] Moussawi K. Kalivas P. W. (2010). Group II metabotropic glutamate receptors (mGlu2/3) in drug addiction. Eur. J. Pharmacol. 639, 115–122. 10.1016/j.ejphar.2010.01.030 20371233 PMC4351804

[B33] Nestler E. J. (2005). The neurobiology of cocaine addiction. Sci. Pract. Perspect. 3, 4–10. 10.1151/spp05314 18552739 PMC2851032

[B34] Quek C. Hill A. F. (2017). The role of extracellular vesicles in neurodegenerative diseases. Biochem. Biophys. Res. Commun. 483, 1178–1186. 10.1016/j.bbrc.2016.09.090 27659705

[B35] Reichel C. M. Moussawi K. Do P. H. Kalivas P. W. See R. E. (2011). Chronic N-acetylcysteine during abstinence or extinction after cocaine self-administration produces enduring reductions in drug seeking. J. Pharmacol. Exp. Ther. 337, 487–493. 10.1124/jpet.111.179317 21303920 PMC3083102

[B37] Scofield M. D. Heinsbroek J. A. Gipson C. D. Kupchik Y. M. Spencer S. Smith A. C.. (2016a). The nucleus accumbens: mechanisms of addiction across drug classes reflect the importance of glutamate homeostasis. Pharmacol. Rev. 68, 816–871. 10.1124/pr.116.012484 27363441 PMC4931870

[B36] Scofield M. D. Li H. Siemsen B. M. Healey K. L. Tran P. K. Woronoff N.. (2016b). Cocaine self-administration and extinction leads to reduced glial fibrillary acidic protein expression and morphometric features of astrocytes in the nucleus accumbens core. Biol. Psychiatry 80, 207–215. 10.1016/j.biopsych.2015.12.022 26946381 PMC4930433

[B38] Trantham-Davidson H. LaLumiere R. T. Reissner K. J. Kalivas P. W. Knackstedt L. A. (2012). Ceftriaxone normalizes nucleus accumbens synaptic transmission, glutamate transport and export following cocaine self-administration and extinction training. J. Neurosci. 32, 12406–12410. 10.1523/jneurosci.1976-12.2012 22956831 PMC3465971

[B39] Xiong Y. Mahmood A. Chopp M. (2017). Emerging potential of exosomes for treatment of traumatic brain injury. Neural Regen. Res. 12, 19–22. 10.4103/1673-5374.198966 28250732 PMC5319225

[B40] Yang Y. Gozen O. Watkins A. Lorenzini I. Lepore A. Gao Y.. (2009). Presynaptic regulation of astroglial excitatory neurotransmitter transporter GLT1. Neuron 61, 880–894. 10.1016/j.neuron.2009.02.010 19323997 PMC2743171

[B41] Zeisel A. Hochgerner H. Lonnerberg P. Johnsson A. Memic F. van der Zwan J.. (2018). Molecular architecture of the mouse nervous system. Cell 174, 999.e1022–1014.e1022. 10.1016/j.cell.2018.06.021 30096314 PMC6086934

